# COVID-19 and social determinants of health: Medicaid managed care organizations’ experiences with addressing member social needs

**DOI:** 10.1371/journal.pone.0264940

**Published:** 2022-03-10

**Authors:** Samuel T. Opoku, Bettye A. Apenteng, Linda Kimsey, Angie Peden, Charles Owens

**Affiliations:** 1 Department of Health Policy and Community Health, Jiann-Ping Hsu College of Public Health, Georgia Southern University, Statesboro, Georgia, United States of America; 2 Center for Public Health Practice and Research, Jiann-Ping Hsu College of Public Health, Georgia Southern University, Statesboro, Georgia, United States of America; UCLA Fielding School of Public Health, UNITED STATES

## Abstract

**Background:**

The significant adverse social and economic impact of the COVID-19 pandemic has cast broader light on the importance of addressing social determinants of health (SDOH). Medicaid Managed Care Organizations (MMCOs) have increasingly taken on a leadership role in integrating medical and social services for Medicaid members. However, the experiences of MMCOs in addressing member social needs during the pandemic has not yet been examined.

**Aim:**

The purpose of this study was to describe MMCOs’ experiences with addressing the social needs of Medicaid members during the COVID-19 pandemic.

**Methods:**

The study was a qualitative study using data from 28 semi-structured interviews with representatives from 14 MMCOs, including state-specific markets of eight national and regional managed care organizations. Data were analyzed using thematic analysis.

**Results:**

Four themes emerged: the impact of the pandemic, SDOH response efforts, an expanding definition of SDOH, and managed care beyond COVID-19. Specifically, participants discussed the impact of the pandemic on enrollees, communities, and healthcare delivery, and detailed their evolving efforts to address member nonmedical needs during the pandemic. They reported an increased demand for social services coupled with a significant retraction of community social service resources. To address these emerging social service gaps, participants described mounting a prompt and adaptable response that was facilitated by strong existing relationships with community partners.

**Conclusion:**

Among MMCOs, the COVID-19 pandemic has emphasized the importance of addressing member social needs, and the need for broader consideration of what constitutes SDOH from a healthcare delivery standpoint.

## Introduction

Most US states use Medicaid Managed Care Organizations (MMCOs) to coordinate the delivery of health services for Medicaid beneficiaries. As of 2018, an estimated 69% of the Medicaid population in 40 states were enrolled in managed care [1]. Evidence suggests that MMCOs can play an important role in addressing Social Determinants of Health (SDOH) [2]. SDOH, which are the non-medical, environmental, and social factors that impact health [3], have a strong influence on healthcare-seeking behaviors, treatment adherence and health outcomes [4–6].

As the health impacts of SDOH have become more apparent, states have also begun encouraging MMCOs to address the social needs of Medicaid beneficiaries as a means to not only improve quality but also lower health care cost. Studies have pointed to unmet social needs as an important contributing factor to rising overall healthcare cost [7], a number that stands at $3.8 trillion or 17% of gross domestic product (GDP) as of 2019 [8]. Accordingly, as of 2020, 35 states had one or more mandates or requirements for MMCOs concerning SDOH, including mandates around screening for social needs, providing referrals to social services, and tracking the outcomes of social service referrals [1]. In response, more MMCOs are attempting to concurrently address the medical and social needs of their members [9]. In a 2018 study of MMCOs, plan representatives identified organizational efforts in areas such as housing, education, nutrition, transportation, behavioral health, and substance abuse [9].

The COVID-19 pandemic has amplified the need to address SDOH. At the height of the pandemic, there were large segments of the country in lockdowns intended to slow the spread of the virus. The proportion of the population with unmet social needs increased significantly as many people became unemployed [10]. Consequently, more individuals became eligible for Medicaid [11]. In March 2020, the number of individuals on Medicaid was estimated at about 71 million people [12]. However, due to the economic effects of the COVID-19 pandemic, by November 2020, that number had increased to about 79 million [13], with states projecting an approximately eight percent increase in Medicaid enrollment in FY2021 [14].

The significant adverse social and economic impact of the COVID-19 pandemic has cast broader light on the importance of addressing SDOH. Data from 211 helplines across the US point to a dramatic increase in the need for social assistance due to the COVID-19 pandemic, including assistance for food and rent [10]. Another study of low-income households revealed an increase in financial instability, employment concerns, food insecurity, housing instability and limited health care access in the wake of the pandemic [15]. Health systems, including those in New York, have also responded to an increased need for food, housing, and financial assistance among their patient population [16].

Experts have identified MMCOs as potential key players in the COVID-19 recovery efforts, particularly in addressing social needs, resource gaps and structural inequities [17]. Accordingly, it is essential to understand their experiences in integrating social and medical services for members during this global crisis. However, there is a dearth of information on the experiences of managed care organizations during the COVID-19 pandemic. For example, a review of the literature published from March 2020 to October 2021 (using the following search terms in EBSCO, CINAHL and Medline databases: managed care, social determinants of health or social needs, and COVID-19) identified only 16 articles which were deemed relevant after title and abstract screening. These articles focused more broadly on the role of the health care industry in addressing social needs and advancing equity following the COVID-19 pandemic. Only two articles [17, 18] focused on managed care’s role in pandemic recovery. To the best of the authors knowledge, no other research study has specifically documented managed care organizations response to social needs of Medicaid beneficiaries during the pandemic. Thus, the purpose of this study was to fill an existing literature gap by describing MMCOs’ experiences with addressing the social needs of Medicaid members during the COVID-19 pandemic.

## Methods

This qualitative study was part of a larger study examining MMCOs SDOH-related activities in eight states (FL, GA, HI, IL, KY, NJ, NY, and SC). Planned data collection efforts, conducted between May 2020 and November 2020, coincided with earlier waves of the COVID-19 pandemic, affording researchers the opportunity to also examine MMCOs’ pandemic response efforts within the context of SDOH. Specifically, the data collection period coincided with the 2020 summer peak in the study states (with the exception of NY and NJ that were two of the earliest states to be affected by the pandemic) and the early phases of the second wave in all eight states.

### Research team

*A* research team consisting of five individuals, two males and three females, with formal training and/or experience in the conduct of qualitative research were involved in data collection, analysis, and interpretation. All researchers are public health faculty with a background in public health and health services administration and research. Interviews and data analysis were conducted in teams of three, with one researcher involved in both data collection and analysis.

### Study sample and recruitment

MMCOs were recruited through purposive and snowballing sampling approaches to participate in key informant interviews. MMCOs serving the eight states (N = 48) were invited to participate in this study. The list of MMCOs by state were obtained from the Kaiser Family Foundation’s Medicaid Managed Care Market Tracker. Point of contact information for MMCOs were obtained through extensive web searches. The invitation letter, sent via email, provided information on the purpose of the study, the research team and funder, the anticipated length of the interview, processes for scheduling an interview if interested, institutional review board (IRB) approval information, and both principal investigator and IRB’s contact information for follow-up questions.

Recruitment efforts were directly targeted at state-specific MMCO organizations, including those affiliated with national plans. Corporate offices of national plans were contacted after failed attempts to contact their state-specific markets. Point of contact information was successfully obtained for 48 out of the 71 MMCOs operating in these states. One MMCO declined to participate, citing time constraints due to pandemic response efforts. MMCOs that agreed to participate identified potential plan representatives who would be able to provide relevant insights on the topic, with some national plans connecting the research team with market (state) specific leadership or teams. Recruitment efforts continued concurrently with data collection and was stopped when saturation was reached. The institutional review board (IRB) at the authors’ institution approved the study.

### Data collection and analysis

Semi-structured interviews lasted, on average 60 minutes and were audio-recorded. Interviews were collected either via telephone or virtual platforms, such as Zoom, depending on participants’ preferences. Prior to the initiation of each interview, participants were read the informed consent and provided with an opportunity to ask questions and have their questions answered. Voluntary participation was stressed. Two consents were obtained and documented in the study’s interview log: consent to participate in the study and consent to audio record the interview. All participants provided verbal consent to both requests, with the exception of one participant who consented to participate in the study but did not consent to the audio recording. For this interview, extensive notes were taken in lieu of the audio recording.

Interviews were transcribed verbatim and coded inductively. Braun and Clarke’s [19] thematic analysis approach was used to analyze the data. As aforementioned, this study was part of a larger study examining MMCOs’ SDOH efforts. Thus, data analysis for this present study focused specifically on participants responses to COVID-specific questions (i.e., *How has COVID-19 impacted your work on social determinants of health*? *How have you modified your strategies to address social determinants of health during the pandemic*?) as well as references made by participants to the pandemic throughout the interviews.

Data analysis began with data immersion, followed by inductive coding, whereby the codes were not specified a priori but rather, were data driven. Two qualitative research-trained researchers independently generated codes based on a subset of the transcripts, after which a consensus codebook was developed and applied to all transcripts. Codes were then collated into emerging themes and subthemes by all three qualitative research-trained involved in the analysis, using a semantic approach that focused on the surface meaning of the data as reported by participants. Themes were subsequently reviewed, revised, and named. All disagreements during the data analysis process were resolved through discussion and consensus-building. NVIVO (version 12) was used for the data analysis.

The goal of the analysis was to present a rich description of the discussions centered on COVID-19 and MMCOs’ COVID-19 pandemic response within the data set. Thus, in line with the expectations of thematic analysis, the study results are presented using a narrative that provides a logical and concise “account of the story the data tell—within and across themes” [19 pg. 23].

## Results

### Characteristics of participating organizations

Twenty-eight semi-structured interviews were conducted with representatives from 14 MMCOs (14/47; 29.8%). These MMCOs included state-specific markets of eight national (N = 5) and regional (N = 3) managed care organizations or health plans, including three of the largest five MMCOs in terms of Medicaid managed care market share in the United States. Collectively, participating organizations represented approximately one out of four MMCOs in the eight states. The average revenue for participating national and regional MMCOs was approximately 39 billion in 2018. Participating MMCOs included an equal mix of for-profit and not-for-profit organizations that had been in operation for at least three decades on average. MMCO representatives who participated in the study included directors or staff of SDOH-focused departments or programs (36%), senior plan executives (25%), medical directors (14%), directors or staff of case management departments (14%) and staff in other roles (11%), including community relations and legal and regulatory affairs (Table 1).

**Table 1:** Participating organizations by state.

	Total	FL	GA	HI	IL	KY	NJ	NY	SC	Multi-state
Participants	28	4	5	2	3	1	6	3	1	3
Unique Organizational Perspective Represented	14	2	2	2	1	1	1	2	1	2
States represented by participants with a multi-state perspective	---	Y	Y		Y	Y	Y	Y		---
% MMCOs in State(s) represented in study^a^	28%	20%	75%	40%	29%	50%	40%	18%	20%	---
**Role of Participants**										
Senior Plan Executive	7	1	0	0	0	0	2	2	0	2
Medical Director	4	2	1	0	0	0	0	1	0	0
Case Management/Care Coordination/Patient Navigation	4	0	2	0	0	1	1	0	0	0
SDOH-Focused Programming	10	1	1	2	2	0	2	0	1	1
Other	3	0	1	0	1	0	1	0	0	0

^a^Computed by dividing the total number of organizations represented in the study by the total number of MMCOs in the state. Participants providing multi-state perspective represented all of their state-specific markets. Thus, they are included in the numerator for each state for which the plan has a state-specific market.

### Emerging themes

Four themes emerged: the impact of the COVID-19 pandemic, SDOH response efforts, an expanding definition of SDOH, and managed care beyond COVID-19. Below we discuss each of these themes and their associated subthemes.

#### Theme one: The impact of the pandemic

Like the rest of the world, MMCOs reported being significantly impacted by the pandemic. The pandemic was described as a chaotic event that mostly caught everyone by surprise.

One participant described their experience as follows:

[S]o when COVID happened, it was an immediate shutdown, all hands-on deck. You knew it was happening, you were watching the news, it was Monday, Tuesday, then it was Wednesday, and the people were like, “Oh, we might have a state of emergency,” and so you’re thinking to yourself as you’re driving home, “Hmm, better go to the grocery store,” you couldn’t find toilet paper, couldn’t find water. Then the companies—everybody is like work from home and do this and shelter in place and oh my gosh, and we were in a pandemic, and we were also in a chaotic atmosphere.

(Participant #16, SDOH Programming Director/Staff, NJ)

Participants discussed the impact of the pandemic on enrollees, communities, and healthcare delivery, in general.

*Subtheme 1*.*1*: *Impact on members*. There was consensus among participants that the pandemic had adversely impacted member health and well-being. Particularly, participants noted an increase in unmet social needs among members. While member needs varied by region and jurisdiction, across MMCOs, food insecurity emerged as the most common social need amplified during the pandemic.

With COVID, I would say the number one thing we have seen is food insecurity. That has probably been the highest. We have done a lot of partnerships with food banks and other areas because as people are losing their jobs, we are seeing that they no longer have access to food.

(Participant #1, SDOH Programming Director/Staff, HI)

Food insecurity has really been the number one issue with COVID-19. So, finding community organizations that will safely deliver food and culturally appropriate food for individuals is something that we’ve been really successful in during COVID-19.

(Participant #20, Other Staff, NJ)

Other emerging member social needs included housing (rental and utilities) assistance, and employment assistance.

Housing support…the CARES Act did not cover every housing entity, and a lot of people were unaware of that, and so now we are experiencing a high volume of calls for rent assistance because people are being displaced or evicted…Of course, jobs. I don’t know if I mentioned that one earlier. Because people—unfortunately, our membership, they may not have the ability to work from home, or some of them may have to go into an actual location, and they may have an underlying condition and so maybe they were unable to keep their job. And so, there was a high, high request for jobs to even now during the pandemic.

(Participant #7, SDOH Programming Director/Staff, GA)

Participants also discussed increased levels of social isolation and emotional distress among members and the need for behavioral and mental health support services.

There was sort of a heightened increase or awareness, or experience with loneliness and being cut off from the outside community.

(Participant #9, Chief Medical Officer, GA)

Little did we know that in 2020, our members were gonna have a high amount of social isolation. So, already having some things that we were looking at for the future, we were really able to implement, not even knowing.

(Participant #6, SDOH Programming Director/Staff, FL)

*Subtheme 1*.*2*: *Impact on communities and community based organizations*. Despite the increased demand for social services, community resources dwindled as the pandemic significantly impacted the viability of the typically small community-based organizations (CBOs) and social services agencies providing these services. Participants spoke at length about the impact the virus has had on community partners and CBOs. CBOs were not only low on resources, such as food and supplies, but they were also short on volunteers since so many people were staying home. Many had to shut down early in the pandemic, leaving vulnerable community members without support.

The community partners that we partner with, a lot of them were struggling before and so with having limited resources, it’s hard to help and provide help to members who need it because your community partners have negative resources.

(Participant #2, Case Management Director/Staff, GA)

Some of the [community] organizations that we had those contracts with, they are closed. They can’t be giving us data and information when they’ve closed, and they’ve closed due to CDC regulations that they can’t uphold. They closed due to their high volunteer base of elderly people that are told to not be out.

(Participant #6, SDOH Programming Director/Staff, FL)

The increased demand for social services coupled with the limited availability of community resources and capacity to meet this demand resulted in significant gaps in social care and unmet social needs:

[T]he resources are just not as available. And there’s so much more need that it’s harder—there’s more people fighting for a limited amount of resources. And those resources are even more limited than they were before. So that’s been a real struggle for us. There have been organizations that are not operating at the capacity that they used to. And that’s really making it a challenge sometimes to find available resources.

(Participant #3, Case Management Director/Staff, GA)

Things are closing. I mean, COVID is causing all types of services, even community-based organizations to close. So now we’re starting to see another set of needs. I think there has been a heightened sense around areas that were being covered that now are not being. I know I’m not explaining this well, but I think overall, we’re seeing more exposure to other social determinants.

(Participant #13, Other Staff, IL)

*Subtheme 1*.*3*: *Impact on healthcare delivery and managed care*. Participants noted that the pandemic had resulted in a significant alteration to healthcare delivery. The utilization of emergency department, hospital inpatient and outpatient services, and primary care provider visits declined, while telephonic and virtual visits increased. One participant noted that while these broader declines in utilization have led to some cost savings, they were most likely temporary savings as some future cost-shifting was expected to occur. According to this participant, the pandemic had created pent-up demand that may eventually increase utilization above pre-COVID levels.

So right now, one of the things all health plans are seeing in every state to my knowledge is lack of utilization, and people aren’t going to the doctor as much. They’re not going to the emergency room as much. They’re just very scared of healthcare systems…[Y]ou [are paid] per member per month if they use care or not. So right now, most plans are kind of building up their reserves because we do feel that there is this pent-up demand for more services. So, we’re not just, you know, socking the money away and not doing anything with it. We’re keeping it in reserve because we know there’s a rush of utilization of necessary services, even the extended benefits and things like that. So just being a good steward to the money you get from the state even in this current environment of low utilization of making sure that you keep it available and not do other things with it.

(Participant #4, Chief Medical Officer, FL)

Despite the general declines in medical care utilization, MMCOs noted that while they were not operating as business as usual, they were still busy addressing members’ overall physical health, mental and social well-being. In direct response to the pandemic, MMCOs altered operational and care processes to adhere to pandemic-related guidelines, such as social distancing. This included a shift to virtual modalities for operating, coordinating, and delivering member services:

[S]o unfortunately right now we’ve had to kind of alter how we conduct our home visits so we’re no longer able to go—at the moment—go out and conduct these home visits like we’re used to… [W]e’re getting creative, doing Zoom calls, to those members that have the capabilities to do Facetime…. We’re actually doing our events through Zoom. So, where we would have our events at a building, we’re doing those through Zoom. We’re doing curbside events. So, people are driving up and doing curbside events. Like one of my community health workers is actually delivering—actually picking up items and dropping off items at the doorstep when we identify members who are needing things. So, that’s kind of how things have kind of altered how we have done business prior to the pandemic.

(Participant #2, Case Management Director/Staff, GA)

Well, I could just say as a company we have taken the state’s requirements, which have been significant, and freed up our providers to do care without a lot of administrative burden. We have gone virtual, both—in pretty much every department. So, if a member has a need we’re doing everything we can to connect through a telehealth environment. And that includes making referrals into social service agencies and community agencies…You know, we have coordination of care still happening. They are happening virtually. So, people are still connected, it’s just not face-to-face.

(Participant #5, Senior Executive, FL)

For several participants, the pandemic did not necessarily change their respective MMCO’s existing SDOH strategy. Instead, it emphasized the importance of investments in this area. The following quote exemplifies the discussions:

As COVID has evolved, we are certainly very focused on where we see the intersection between social need and medical [need]…I think this is actually almost kind of the exclamation point on exactly why we’re doing it [addressing social needs], and we need to—and I would argue within [ours] and other businesses, it’s the time to double down, not the time to pull away. I think we’re gonna learn a lot after COVID on what we could have done better.

(Participant #25, Senior Executive, National/Multi-state)

#### Theme two: MMCOs’ SDOH response efforts

The COVID-19 pandemic has led MMCOs to pivot, and re-work plans to respond to the pandemic effectively. Participants described an urgent yet adaptable organizational pandemic response that required creativity and grassroots community mobilization. MMCOs noted their ability to respond quickly, yet meaningfully, to the pandemic was facilitated by strong existing community relationships and data-informed strategic prioritization. The following quotes exemplified these expressed sentiments:

In regard to COVID-19, health plans were charged with trying to figure out how to support the membership that we had in places that we were already working in. And the thing is that, as a Medicaid health plan, we were dealing with the most vulnerable populations that you can deal with, which gave us a charge to actually do this and to look at trying to figure out how this could work and what the investments could be. [We were] trying to be strategic, again, utilizing data where it could be [and] utilizing organic relationships where we had community connections. We had feet on the street already. We had relationships built. We know the organizations…And the combination of all of that is where it allows us to be a little bit more nimble, organic in each market to be able to invest dollars quickly to the organizations that needed it as fast as possible because we were already addressing social determinants…So, I think we were really in position to be able to impact and help I think at a quicker rate

(Participant #14, SDOH Programming Director/Staff, IL)

It was only because this [MMCO SDOH] program existed for so long and already had established those relationships that [we] met those needs of 100 members every two weeks, finding partners whom we would call, whom we knew. We knew cell phone numbers, we knew where people were at, "Hey, who’s got a program? Okay, ‘in [city] this particular agency is shut down, but this one’s open or opening up in this state’. We were making resource guides; we were not only giving members referrals, we were [also] coordinating: ‘Okay, I’m on one phone. So and so is coming to the house to deliver the bag. Okay, let me call the member.’ We were only able to do that because of the relationships of this program. So, that’s impact in and of itself.

(Participant #16, SDOH Programming Director/Staff, NJ)

Response efforts centered broadly on directly assessing and addressing member needs and expanding community capacity to address gaps in social services.

*Subtheme 2*.*1 Directly addressing member needs*. MMCOs pandemic response efforts included direct outreach to members to meet both medical and non-medical needs. Member outreach activities were informed by COVID-specific needs assessment and deployed largely via virtual modes of communications.

So, if a member has a need, we’re doing everything we can to connect through a telehealth environment. And that includes making referrals into social service agencies and community agencies.

(Participant #5, Senior Executive, FL)

We’ve got something we call [name of App], which is our integrated member experience platform—It’s the call center, field operations, provider services, health services. So, every time a member makes an inquiry via the app, they’re automatically funneled to our integrated care team for intervention and care team connect is also able to identify needs based on claims data that gets integrated with the system and then pushed down automatically to our care team.

(Participant #26, Senior Executive, NY)

In addition to assessing COVID-19 related member needs, MMCOs described creating or updating a directory of COVID-19 resources to help members identify community resources for varying social and medical needs:

[I]mmediately at the onset of COVID-19, the [SDOH] Department immediately revved up and created on a weekly basis a resource guide to track what resources are available; it was a statewide resource guide. We sent it out to our community partners. We also ensured that our members [had it]. We sent it out to the whole organization.

(Participant #15, SDOH Programming Director/Staff, SC)

We already have a pretty extensive list of community-based organizations, but we, in all of our regions, went back and expanded that list to cover as much of the need that there was.

(Participant #22, Chief Medical Officer, NY)

Anyone can go to any of our different branded sites and use our vendor to find by ZIP code, easily, free, or low-cost COVID assistance in your community, whether it be ‘where’s a local food pantry?’ ‘What’s the number to get the SNAP and WIC office to see if I’m eligible for the food assistance program through the USDA?’ Or ‘here’s something that can help me pay my electricity bill or even my rent for the next coming months’.

(Participant #25, Senior Executive, National/Multi-site)

MMCOs also worked with community partners to provide direct outreach and linkage to resources. MMCOs implemented these activities, even within the context of dwindling community social service resources, as previously described, and as a result often had to come up with innovative solutions to address the unmet social needs amplified by the pandemic:

So, we’ve had to partner. All of the MMCOs have collectively gotten together. We’ve done fliers that really represent the services that each of the health plans can provide to their members. We’ve worked with food banks or shelters where they’ve inserted these fliers to distribute to various individuals to say, ‘if you need help with the following, please call this number and we will help you’.

(Participant #1, SDOH Programming Director/Staff, HI).

Another example of how we had to really pivot and shift quickly: one of our key partners—we’ve been involved with them for several years since we started the program in [our state], and initially, as a partner, we would go to their organization and get diapers and baby items and take them to our members; so our social workers would do that. Of course, because of social distancing, things of that nature, our teams could not go out and do that. So, we had to work with [our partner] and say, ‘What do we need to do to create a solution so that our moms are getting diapers and they’re getting baby items because they’re at home even more now during this pandemic?’ And so we really leveraged our partnerships with food banks in the area. Most of these parents were going to the food bank, and so, [this partner] now delivers the diapers to the food banks. So, when they’re getting their food supply, they can now pick up diapers from the food bank.

(Participant #7, SDOH Programming Director/Staff, GA).

*Subtheme 2*.*2 Expanding community capacity*. There was consensus among participants that the ability to effectively address the social needs of members is hinged on the capacity of community-based organizations and partners. Participants noted that community response efforts required MMCOs to have a pulse on the community. Knowledge of community needs during the pandemic was obtained through direct embeddedness within the community, and consultations with community partners. Participants shared some creative ideas for supporting community partners and community-based organizations to meet community needs, including supporting CBOs financially through donations and grantmaking activities. Participants shared that they were able to provide direct donations, scholarships, and grants to community groups and local foundations who have “*their finger on the pulse of the need*” of the community. These funds supported, among others, youth resource centers, personal protective equipment purchases, food, and food delivery, and WIFI. Through this support, MMCOs helped expand the capacity of CBOs to serve not only their members but the community at large. The following quotes exemplified this emerging subtheme:

When the pandemic hit, we knew that a lot of them [CBOs] would be running out of resources, but we knew that a lot of our members went there. So, when we had the opportunity to give away gift cards, like 200 gift cards, we immediately gave gift cards to them…because they had done so much for our members.

(Participant #2, Case Management Director/Staff, GA).

We’ve provided funding for food distribution during the middle of COVID. For example, the [name of community-based agency] was doing “grab and go” breakfasts and lunches for kids who depended on breakfast and lunch at public school, and now with COVID, they didn’t have schools to go to. The [agency] had up to eight locations to provide breakfast and lunch, and they were, of course, kind of shutdown so their revenue side wasn’t really coming in.

(Participant #10, SDOH Programming Director/Staff, HI).

#### Theme three: Expanding definition of SDOH

Participants described an evolution in the way their organization defined and responded to social determinants of health. Participants noted that the pandemic had expanded their “working definition” of SDOH as well as who they considered targets for SDOH related activities.

*Subtheme 3*.*1 Expanded definition of SDOH*. MMCOs described observing members’ social needs that they had previously not considered or addressed. They noted that the pandemic had led to an increased recognition of the digital divide as an important determinant of health that required urgent attention. Other emerging non-traditional SDOHs (in terms of what MMCOs typically address) included racism and racial inequities, childcare, and employment assistance.

The following quotes summarized these discussions:

A lot of people are needing assistance because they’re homeschooling their children. A lot of people are needing assistance with Wi-Fi access and computer needs, things like that. So, that is something that is very new to us because we’ve never had anyone call in to say that they need assistance or help with—paying assistance with a computer, or we had someone call in who needed—they were renting a computer, and it was about to be repossessed, and school was about to start so they needed assistance with attempting to try to keep their laptop for that child. So, it’s been different things, and it’s been really kind of all over the place.

(Participant #2, Case Management Director/Staff, GA).

In some realms, people would say having the Internet is a luxury. Now, having the Internet is a necessity. Previously, people may have thought the Internet is not a need, but now with COVID, it is. So social determinants definitely have expanded.

(Participant #13, Other Staff, IL).

[R]acism also must be addressed as a social determinant of health. The COVID-19 pandemic has shone a bright light on the devastating effects of disparities in communities of color… The health inequities exist because of serious structural barriers to accessing quality and affordable healthcare, rampant discrimination, and higher rates of poverty.

(Participant #28, Senior Executive, NY)

*Subtheme 3*.*2 Expanded targets for SDOH efforts*. There was an acknowledgment that COVID-19 was a pervasive determinant of social need, which led some MMCOs to expand the target population for SDOH efforts to include populations not traditionally considered “socially vulnerable”, such as enrollees in their commercial business or their own employees.

COVID-19 was just another social determinant. And unlike all the other social determinants, this was one that everyone had to deal with.

(Participant #14, SDOH Programming Director/Staff, IL).

Look at what’s happening with Covid today. We have 30 million people that are unemployed and lots of people that are hurting. A lot of those members, a lot of those people from our commercial side of the business, are now gonna be coming over and then applying for Medicaid in our government business…So we need to be doing things on the commercial side of our house that we’ve learned from the things that we’ve done in the Medicaid business. We can now take these strategies to some of our commercial customers that, frankly, have pockets of their populations that look more like a Medicaid population than the commercial side. Think of folks that are low wage earners…those populations need this kind of help. They need food strategies to help them as well.

(Participant #25, Senior Executive, National/Multi-site)

#### Theme four: Managed care beyond COVID-19

Participants noted that the effects of the pandemic were likely to be long-term in many aspects of healthcare delivery. While the pandemic has significantly impacted health and well-being, participants noted that there were some silver linings emerging from the experience. The pandemic had promoted an openness to change, and some felt these changes were for the better:

I think a lot of us feel that the way we do business, the way the state does business, a lot of things are going to change because of COVID. Because we’ve learned things about how we can do things differently and how we can do things better.

(Participant #18, Senior Executive, NJ)

Notable among the changes was the increased recognition of the importance of SDOH, the growth in healthcare communication technology and changes in healthcare delivery, and the expansion of existing community networks.

*Subtheme 4*.*1*. *Increased recognition of the importance of SDOH*. There was a consensus that the pandemic had cast SDOH into the limelight, resulting in a broader recognition, among stakeholders, of the interconnections between social, physical, and mental health. It had also become clearer that social needs often did not occur in a vacuum. Individuals’ social needs are related in a more complex way and the pandemic reflected this. One MMCO mentioned observing this domino effect with their membership, where one social need led to another:

At first, it was food. At first, it was some of our highest gaps…My team usually did around 20 gaps a month. Come about March, my team was doing almost 200 gaps a month. Gaps mean they couldn’t find anything in the system open, the case management, when they were trying to help…When you think about several hundred gaps per month of trying to find organizations, most of those at first were food. That’s where it hit the most. Then, after the food—that was usually through March, April, May—come June, July, we started seeing a large influx of financial assistance: rent, utilities, mortgage. They were looking for unemployment [assistance]. Those were our huge ones, where they were looking for some kind of job…When you think about the hundreds of thousands of people without work, definitely right now it’s financial assistance.

(Participant #6, SDOH Programming Director/Staff, FL).

This increased recognition about the magnitude of unmet social needs and its associated health impact was likely to spur on changes in expectations concerning how care is delivered. As two participants stated:

In light of COVID, I think there’s just gonna be more of an openness in general that the system clearly failed a lot of people, and the most vulnerable among us most significantly… I think that COVID will give us this opportunity, whenever it calms, to maybe just rethink Medicaid, and healthcare in general, just a little more broadly.

(Participant #20, Other Staff, NJ).

I think this is an opportunity in our country to change the way that we approach social services and the way that everybody is involved. You talk about the expectations of MCOs. I think this pandemic has changed the expectation of everybody, clinical systems, MCOs, community, the government, everybody.

(Participant #24, SDOH Programming Director/Staff, National/Multi-state).

Another described the potential impact the pandemic will have on the managed care industry, specifically:

Managed care as a whole—like I said I think [the pandemic] will definitely make us more collaborative in thinking about people’s social well-being and their physical health. How we collaborate with providers, and you know on the operational manager side, about all of those things. It’s just making us look at everything and how we kind of do business, period.

(Participant #2, Case Management Director/Staff, GA).

MMCOs expected more state regulations or expectations of MMCOs concerning addressing member social needs, with some even remarking that states had already been asking them to do more on the SDOH front as part of the pandemic response.

[N]ow that the state has begun to express more interest in learning about the impact of social determinants of health that helps too. Because when the states begin to send questions and wanting to know ‘what are you doing for this?’ that means that the conversation is now occurring at a state level, which means hopefully there will be some policies around things and some standardized practices around social determinants.

(Participant #7, SDOH Programming Director/Staff, GA).

*Subtheme 4*.*2*. *Growth in healthcare communication technology and changes in healthcare delivery*. Participants felt that the pandemic had spurred growth in healthcare technology. There was a consensus that innovations in virtual healthcare technologies and delivery modalities were positive and here to stay:

I think virtual visits for some office visits is going to be standard now for those basic visits. I don’t think those will ever go away.

(Participant #2, Case Management Director/Staff, GA).

Issues like telehealth and virtual health are really being ramped as one of the outcomes that will last on [beyond] COVID.

(Participant #25, Senior Executive, National/Multi-site)

*Subtheme 4*.*3*. *Expanded community networks*. For some MMCOs, pandemic response efforts had led to an expansion of their existing community networks as they have had to forge new relationships with community partners. Virtual modalities for engaging community partners had also removed geographical barriers and allowed them to expand their reach and connection with community partners.

Participants described this as follows:

What has happened through COVID, which is a beautiful thing, is a lot of new organizations are opening. So, we are learning about new organizations…Yes, many, many have closed, but there are a lot of new organizations that we’re getting to know to date. So, one of the beauties of COVID is it’s bursting out these organizations that are now really taking a stronger stance and a lens on these gaps that we’ve been talking about. Just like with all of the health inequities, the racial inequities, COVID has really put a microscope on all of those things that impact healthcare access and social determinant access. So that’s what’s happening. We’re learning and new organizations are being birthed out of this, so I think it’s phenomenal because of that.

(Participant #7, SDOH Programming Director/Staff, GA).

In a virtual world, you know, working with COVID-19, a lot of places have had no choice but to go virtual. So that kind of helped us out in terms of travel in distance, you know, covering a great distance…It was interesting that a lot of these new [community] relationships came from rural communities. You know, in which they did not have resources such as food pantries that someone could go down the street. There were different agencies that were delivering meals to homes for senior citizens…the social isolation, you know, with people not going out. It allowed us to basically cast a net and we found an opportunity to get in the nooks and crannies that we literally probably would have almost overlooked in any other time before COVID-19.

(Participant #15, SDOH Programming Director/Staff, SC).

## Discussion

The purpose of this study was to describe the experiences of MMCOs in addressing member social needs during the COVID-19 pandemic. Consistent with previously reported studies [10, 20], MMCO representatives participating in this study reported a dramatic increase in unmet social and mental health needs of Medicaid members, which resulted in an increased need for social services. Food insecurity was cited as the most commonly occurring need, consistent with data from 211 helplines across the US [10]. In a similar study assessing the SDOH-related needs of low-income households with children during the pandemic, about two-thirds of survey respondents reported concerns about food availability [15]. This immediate and significant need for food support differed from earlier stated priorities of MMCOs, where housing was the primary focus [9].

The COVID-19 pandemic has been described as an accelerant of changes in several areas of healthcare, including remote work, telehealth, diagnostics, and vaccine development: trends that had been underway are believed to have advanced substantially because of the pandemic [21]. This same concept of pandemic-driven advancement was described by participants in the area of SDOH. For accelerants for change to have direction and be meaningful, strategic initiatives need to be underway. In the area of SDOH, those MMCOs that had a well-developed organization and system of service provision felt strongly that their previous efforts prepared them to be able to adapt and to address the many significant social needs that quickly arose as the COVID-19 pandemic took hold. Previously established community relationships provided a foundation that enabled MMCOs to sustain their SDOH efforts. Community partnerships have long been identified as the bedrock of MMCOs’ efforts to meet the needs of the vulnerable populations and communities they serve [22]. Community-based social services organizations are important partners in efforts to holistically address member medical and non-medical needs, given managed care organizations’ lack of direct expertise in addressing nonmedical needs [23]. The pandemic experiences, shared by participants in this study, highlighted the need to recognize CBOs and community-based social service agencies as important partners in healthcare delivery, and lent support to existing calls for the formal integration of social care into health care delivery [24].

The findings from the study also suggest that the pandemic became a focusing event for MMCOs. For some, the pandemic served to confirm that their past efforts in SDOH had been productive and worthwhile. COVID-19 also focused new light on other lesser-known social determinants of health, like social isolation, disparities in internet access and racism. In addition, the broader social impact of COVID-19 on many individuals also led some MMCOs to reconsider and broaden their existing definition of “target populations” for their ongoing SDOH related efforts. Collectively, these findings echo the ongoing calls for a broader, and more direct consideration of social determinants of health, following the pandemic [25]. Recent studies have identified the need for an expanded view of SDOH, including a more focused examination of structural racial inequities and health disparities [26]. Further, with the pandemic-driven shift to virtual modalities of health—which has been described as a “new-normal” and is expected to stay [27]–attention has also been drawn to the potential adverse impacts of the digital divide on health-seeking behavior and health outcomes [28, 29].

Overall, MMCOs’ commitment to addressing SDOH among their members is reflective of a widespread recognition of the important influence of non-medical factors on health outcomes. States, an important stakeholder, were beginning to prioritize SDOH even before the pandemic and had become more interested in using managed care as a vehicle for addressing SDOH among the Medicaid population [30]. While participants in this study felt states’ efforts concerning SDOH would accelerate due to the COVID-19 pandemic, it remains to be seen how the pandemic will shape state expectations of MMCOs concerning SDOH, moving forward.

## Limitations

This study’s examination of the pandemic-related experiences of MMCOs is timely and relevant, especially given the increase in unmet social needs and MMCOs’ increasing role in addressing the nonmedical needs of Medicaid members—a significant proportion of whom were considered socially vulnerable even before the pandemic. Despite its strengths, however, the study is not without limitations. As with all qualitative approaches to inquiry, there are limitations to the extent to which the study’s findings can be generalized to participants beyond the study’s sample. The data collection period coincided with the early pandemic response efforts, thus limiting MMCOs’ participation rates. Perspectives obtained were representative of only approximately one out of four MMCOs serving in the eight states examined in this study. Further, the use of purposive and snowballing sampling approaches limits the extent to which the study’s findings can be generalized to all MMCOs. Additionally, due to significant researcher interaction with participants, there is a risk for researcher effects and social desirability bias in qualitative research studies. It is possible that MMCOs may have misrepresented or overstated their ongoing SDOH efforts. We were unable to objectively characterize the level of social needs of members served by participating organizations or objectively quantify organizations’ efforts to address SDOH. As such, the authors had no way of verifying any of the information provided by the participants. However, the consistency of the findings with the existing literature provides support for participants’ accounts.

## Conclusion

The study described the experiences of MMCOs in addressing member social needs during the COVID-19 pandemic. Study participants described the impact of the pandemic on enrollees, community partners, MMCOs’ operations, and SDOH-related efforts, and on healthcare delivery. Participants also noted areas in which COVID-19’s significant impact is expected to continue. According to participants, the pandemic had (a) increased stakeholder (including States) recognition of the importance of addressing SDOH, (b) spurred significant growth in the use of healthcare communication technology to improve connectedness with both beneficiaries and CBOs, (c) transformed healthcare delivery because of large shifts in workload from in-person settings to virtual ones, and (d) expanded and enriched existing community networks for addressing SDOH. These trends are expected to shape the environment of MMCO-provided SDOH going forward. To adapt to these trends, greater MMCO collaboration—between social and clinical care functions, and even across the silos of Medicaid and commercial managed care businesses may be warranted. For MMCOs, the crucible of COVID-19 has energized efforts in the provision of SDOH. As one participant emphasized, *it is time to double down and not pull away*.

## Supporting information

S1 File (DOCX)
