# Supplementary material for: COVID-19 and social determinants of health: Medicaid managed care organizations’ experiences with addressing member social needs
Source: PLoS One. 2022 Mar 10;17(3):e0264940. doi: 10.1371/journal.pone.0264940 (PMC8912251; doi:10.1371/journal.pone.0264940)
Supplement: S1 File — (DOCX) [file pone.0264940.s001.docx]

Note: This represents the interview guide for the larger study. Present study focuses only on COVID-19 discussions (primarily Q16-Q17)

**Interview Guide for Medicaid Managed Care Organizations**

*[Read and Review Informed Consent]*

*Do you consent to participate in this study?*

*May I please have permission to audio-record this interview for later transcription?*

*Let’s begin…*

**Your Organization**

1. Please tell me about your organization and the work you do

[Probes: In total how many states do you serve? How many states do you have Medicaid contracts with?]

**Your Organization’s Effort to Address Social Determinants of Health**

1. Please describe your organization’s position with respect to addressing social determinants of health within the Medicaid population.
   1. Why is it important for managed care organizations (MCOs) to address SDOH of their enrollees?

[Probe: What would you say are some of the drivers motivating your own MCO to address Medicaid enrollees’ social needs?]

1. What are the activities being implemented by your organization to address social determinants of health within the Medicaid population?

[Probes: How are these efforts structured? Where do you look for guidance and support on how to design effective programs? How do you tailor your approaches to suit the needs of complex patients?]

**Partnerships to Address Social Determinants of Health**

1. Please describe the partnerships you have developed to address SDOH of Medicaid enrollees.

[Probes: What types of organizations do you partner with most commonly? How do you manage and sustain the partnerships you have described?]

- 1. To what extent do you engage community-based organizations (CBO) in addressing social needs of Medicaid enrollees?

[Probes: How do you engage them? What do these partnerships look like, what types of CBOs are involved, and how are CBOs paid for the services they provide?]

1. What has been the impact of these partnerships?

[Probes: What is the impact on Medicaid enrollees’ health care costs and utilization, social service utilization, and health outcomes? What is the impact on the capacity and finances of the CBOs involved?]

1. Effectively addressing social determinants of health may require information and resource sharing among partners and stakeholders. How does your organization exchange social needs and utilization data among stakeholders, and partners, including community-based organizations and the state?

**State Expectations and MCO SDOH Programming**

1. How do your approaches to address social determinants of health among the Medicaid population differ from state to state?

[Probes: What factors influence your approach in a given state? Have you been able to implement lessons learned in one state across others?]

1. Please describe any state-specific expectations concerning how your organization and other MCOs address the social needs of Medicaid enrollees.

[Probes: Do they use contracts to encourage MCOs to address social determinants of health? Do they use reimbursement mechanisms or other financial incentive to encourage MCOs to address social determinants of health? What other strategies do they use to encourage MCOs to address social determinants of health?]

1. How do state expectations (or the lack of expectations) influence your approach to addressing social determinants of health within the Medicaid population?

**Assessing the Impact of Social Determinants of Health Investments**

1. How does your organization assess the impact of the investments or efforts to address Medicaid enrollees’ social needs?

[Probes: How do you determine if your efforts are impacting health outcomes? How does your MCO recognize a return on investment in relation to these activities? What processes are used to determine whether MCOs’ activities are appropriate and responsive to community needs? To what extent are the perspectives of enrollees included in such evaluations of impact?]

1. In general, to what extent does your MCO solicit input from enrollees, advocates and other stakeholders to inform their cost containment strategies and patient outcomes?

**Challenges and Opportunities in Addressing Social Determinants of Health**

1. What are the challenges associated with your implemented approaches to address the social determinants of health within the Medicaid population?

[Probes: How is your organization approaching and addressing these challenges?]

1. In addressing enrollees’ social needs how does your MCO address challenges and opportunities related to data, data systems, data sharing with state Medicaid offices?

[Probes: How is your MCO able track enrollee referrals, costs and service utilization in sectors outside of health care? How granular is your MCO able to obtain these data? What are promising solutions to common data challenges?]

1. What are additional opportunities for addressing social needs of Medicaid population?

[Probes: What are your organization’s plans for taking advantage for acting on these opportunities in the future?]

**Sustainability of Social Determinants of Health Investments**

1. How financially sustainable are your current investments or efforts to address social determinants of health?
   1. How do you ensure the sustainability of your existing investments or efforts to address social determinants of health?

[Probes: How will environmental dynamics, such as economic downturn shape your efforts?]

**COVID-19**

1. How has COVID-19 impacted your work on social determinants of health?
2. How have you modified your strategies to address social determinants of health during the pandemic?

**Conclusion**

1. Given your organization’s past and current efforts to address the SDOH of Medicaid and other populations, what would you describe as lessons learned or best practices for such efforts?
2. Are there any other areas that we failed to touch on, that you would like to discuss?

*Thank you for your time. We appreciate the invaluable information that you have shared with us. Thank you for all you do to promote health equity and improve the health outcomes of underserved populations.*
